# Episodic memory assessment: effects of sex and age on performance and response time during a continuous recognition task

**DOI:** 10.3389/fnhum.2024.1304221

**Published:** 2024-04-04

**Authors:** James O. Clifford, Sulekha Anand, Franck Tarpin-Bernard, Michael F. Bergeron, Curtis B. Ashford, Peter J. Bayley, John Wesson Ashford

**Affiliations:** ^1^Department of Psychology, College of San Mateo, San Mateo, CA, United States; ^2^Department of Biological Sciences, San Jose State University, San Jose, CA, United States; ^3^HAPPYneuron, Inc., Lyon, France; ^4^Department of Health Sciences, University of Hartford, West Hartford, CT, United States; ^5^MemTrax, LLC, Redwood City, CA, United States; ^6^CogniFit, LLC, Redwood City, CA, United States; ^7^VA Palo Alto Health Care System, Palo Alto, CA, United States; ^8^Department of Psychiatry and Behavioral Sciences, Stanford, CA, United States

**Keywords:** memory, episodic memory, dementia, cognitive assessment, response time

## Abstract

**Introduction:**

Continuous recognition tasks (CRTs) assess episodic memory (EM), the central functional disturbance in Alzheimer’s disease and several related disorders. The online MemTrax computerized CRT provides a platform for screening and assessment that is engaging and can be repeated frequently. MemTrax presents complex visual stimuli, which require complex involvement of the lateral and medial temporal lobes and can be completed in less than 2 min. Results include number of correct recognitions (HITs), recognition failures (MISSes = 1-HITs), correct rejections (CRs), false alarms (FAs = 1-CRs), total correct (TC = HITs + CRs), and response times (RTs) for each HIT and FA. Prior analyses of MemTrax CRT data show no effects of sex but an effect of age on performance. The number of HITs corresponds to faster RT-HITs more closely than TC, and CRs do not relate to RT-HITs. RT-HITs show a typical skewed distribution, and cumulative RT-HITs fit a negative survival curve (RevEx). Thus, this study aimed to define precisely the effects of sex and age on HITS, CRs, RT-HITs, and the dynamics of RTs in an engaged population.

**Methods:**

MemTrax CRT online data on 18,255 individuals was analyzed for sex, age, and distributions of HITs, CRs, MISSes, FAs, TC, and relationships to both RT-HITs and RT-FAs.

**Results:**

HITs corresponded more closely to RT-HITs than did TC because CRs did not relate to RT-HITs. RT-FAs had a broader distribution than RT-HITs and were faster than RT-HITs in about half of the sample, slower in the other half. Performance metrics for men and women were the same. HITs declined with age as RT-HITs increased. CRs also decreased with age and RT-FAs increased, but with no correlation. The group over aged 50 years had RT-HITs distributions slower than under 50 years. For both age ranges, the RevEx model explained more than 99% of the variance in RT-HITs.

**Discussion:**

The dichotomy of HITs and CRs suggests opposing cognitive strategies: (1) less certainty about recognitions, in association with slower RT-HITs and lower HIT percentages suggests recognition difficulty, leading to more MISSes, and (2) decreased CRs (more FAs) but faster RTs to HITs and FAs, suggesting overly quick decisions leading to errors. MemTrax CRT performance provides an indication of EM (HITs and RT-HITs may relate to function of the temporal lobe), executive function (FAs may relate to function of the frontal lobe), processing speed (RTs), cognitive ability, and age-related changes. This CRT provides potential clinical screening utility for early Alzheimer’s disease and other conditions affecting EM, other cognitive functions, and more accurate impairment assessment to track changes over time.

## Introduction

Aging affects learning, memory, and cognitive function, and the number and proportion of elderly individuals with various levels of cognitive dysfunction are rapidly increasing (Gbd 2019 Dementia Forecasting Collaborators, 2022; Singh et al., 2023; Chithiramohan et al., 2024; Malzbender et al., 2024). Cognitive disorders, from mild impairment to severe dementia, are caused by a variety of conditions from Alzheimer’s disease (AD) to cerebrovascular diseases, the sixth and fourth leading causes of death in the United States, respectively in 2023^1^. In these conditions, cognitive impairment is characterized predominantly by deterioration of episodic memory (EM), defined here as a type of long-term memory (LTM) for previous experiences, such as an item or event which persists for minutes, hours, days, or a lifetime, through distracting occurrences. In most dementias, EM function deteriorates over several years, so those affected forget gradually and progressively more information after a few minutes have passed. In fact, accelerated forgetting after distraction is probably the best predictor of cognitive decline (Wearn et al., 2020), reflecting concurrent medial temporal lobe atrophy (Marizzoni et al., 2019), the region of the brain directly responsible for initiating EM. A variety of acute insults affecting the medial temporal lobe, including traumatic brain injury and hypoxic encephalopathy, can cause sudden and permanent impairment of EM. Thus, there is a pressing need to develop effective and efficient tests to determine the level of EM impairment for medical and social assessments and interventions. With the ever-increasing implementations, applications, and utilizations of computers and online systems, cognitive assessment can be performed quickly and cost effectively (Sabbagh et al., 2020; Chan et al., 2021; Ding et al., 2022), including tests which are “culture-fair” (Chithiramohan et al., 2024) and use analysis with machine learning techniques (Bergeron et al., 2019, 2020; Nwanosike et al., 2022). One such test for this purpose, MemTrax (Ashford et al., 2019), was developed for screening of cognitive impairment specifically related to EM dysfunction. The MemTrax platform has proven to be engaging with over a million individuals having taken the test across several countries, with many users taking it more than 100 times and some over 1,000 times (Ashford et al., 2022a).

### Signal detection tasks and continuous recognition tasks

Measurement of learning, memory, and related cognitive functions frequently relies on signal detection tasks (SDTs). One type of SDT instructs a participant to attend to all task stimuli and detect specific “target” stimuli (Goldstein et al., 2019). In such SDTs, the user is instructed to attend to the screen which provides the stimuli, and the task can last for a prolonged period, i.e., over many minutes or even hours. The instructions for such SDTs must reside in working memory (WM, managed by attention and executive systems) while the “target” stimuli reside in limited-capacity, modality-independent short-term memory (STM, which can only retain a limited number of items of information for less than a minute without distraction). These SDTs do not significantly assess EM because the instructions and “target” stimuli are constantly refreshed during the task.

Information processing models (IPMs) account for the events that occur in the brain of the participant during such SDTs, with the initial neural processing of each new stimulus conceptualized as occurring in sensory memory (SM, lasting only a few seconds) (Sperling, 1963). The timing and accuracy of behaviors performed in response to the “target” stimuli during the task are the metrics of interest. These measures reflect the effort expended by those brain events engaged when the electrical representations of the stimulus reach the massive capacity and modality-dependent cortical region. In SDTs where there are predetermined target stimuli and non-target stimuli, each stimulus occurs and is represented within the STM of the defined target specified for processing (Sternberg, 1966). These STM representations interact with processes as directed and instructed to operate in WM (Baddeley, 1982) to determine if the newly presented stimulus matches a “target” or not. At the neurophysiological level, neural systems compare each newly presented stimulus to neural representations in the assigned cortical region of the target stimulus using the neuronally encoded criteria in STM to decide whether the new stimulus matches the designated target and is recognized (Ashford et al., 1998). In these tasks, the level of function of STM can be gauged by the number of stimuli set as targets at the beginning of the task (Sternberg, 1966).

The continuous recognition task (CRT) is a different testing approach within the SDT category and has become a standard approach for assessing EM (Hockley, 1982, 2022). In a CRT, stimuli are presented in a series, and the participant is instructed to attend to each stimulus and indicate if a stimulus is a repetition of one previously shown in the series. If the stimulus is new, the participant is instructed to encode the new item. With ongoing stimuli, the number of items quickly exceeds the capacity of STM. Consequently, new items much be transferred into EM for later access. Accordingly, a CRT is an important approach to EM assessment.

### Neural processing of information and episodic memory in a CRT

When a stimulus is presented in a CRT, its representation in brain neural networks must be compared with representations which were previously presented. However, the number of stimuli presented rapidly exceeds the capacity of STM. Accordingly, the IPM must be revised to consider that the neural representation of the new stimulus must be compared to the integrated representations of previously processed information in massive-capacity and modality-independent EM. This comparison may elicit a recognition response in the brain’s processing system, indicating a repeated, image, that is, a “target.” If there is no perceptual recognition of the new stimulus (i.e., the stimulus is not recognized as repeated), the information about the new stimulus, now residing in STM, will be transferred into EM for inclusion in analyses of subsequent trials. Later in the test, these neural processes can retrieve information about this stimulus from all previously presented stimuli residing in EM for analysis (Baddeley and Hitch, 2000; Baddeley et al., 2019). Newly presented stimuli will each be compared to previously presented stimuli to determine whether it is a repeated (“target”) image.

Whereas the discussion of WM, STM, and EM provides a rubric for understanding the psychological processes constituted by the brain’s neural systems, the critical issue is that the neural systems process information in a parallel and massively reciprocal fashion across networks containing billions of neurons (Hawkins and Ahmad, 2016; Sherwood et al., 2020), and when information moves from STM into EM, the pattern of activation of trillions of synapses is analyzed. If the pattern is recognized during a CRT, the instructed action is performed, but if the pattern is not recognized, then a neurochemical activation is initiated to establish the new pattern in the neural network (Ashford et al., 2011a). It is this latter activation, associated with the fundamental neuroplasticity of the brain, that is the important component tested by the MemTrax CRT described here (Ashford, 2023).

Signal Detection Theory provides empirical and analytical methods for exploring factors suggested to alter information processing during a task (Massaro and Friedman, 1990). One factor is the participant’s internal state. Other factors are external events not related to the task. These factors alter the quality and distinctiveness of the representation persisting in each memory construct, specifically the activation states of the recruited neural networks and the sensitivity for engagement of the information processing apparatus. These theoretical constructs are relevant to explain the central neural processing that occurs during specific cognitive assessment tests, in this case, a CRT, but there is a question about whether the specific mathematical models associated with this theory (*d*′ and beta) are applicable when new stimulus information is constantly being added to the array of items already presented.

The processes involved in the management of CRT stimuli are responsible for the timing and accuracy of the decision that a stimulus matches the designated “target” that the individual is instructed to recognize and so indicate by a response. In the CRT, one of the four behavioral options to a presented stimulus occurs, a HIT (user recognizes the pattern of the presented stimulus as a match and responds with the prescribed action), a correct rejection (CR, the user does not recognize the presented stimulus since the stimulus had not been previously shown, and the user does not respond), a MISS (user fails to recognize the repeated presented stimulus and the user does not respond), and a false alarm (FA, user falsely perceives the presented stimulus as a repeat, and the user responds inappropriately with the prescribed response). The HITs and CRs can be summed to provide the total correct (TC) trials. For every presentation, timing is recorded from the onset of the stimulus until the activation of the sensor or 3,000 ms. Response time for either HITs or FAs is recorded as the number of milliseconds from the onset of the stimulus to the activation of the sensor (e.g., spacebar) by the user, and the next stimulus is shown immediately. If the sensor is not activated by 3,000 ms, the next stimulus is shown at that time. Responses and non-responses are noted according to the presentation order to tabulate HITs, FAs, CRs, and MISSes, and at the end, the average RT-HITs and RT-FAs is calculated. Numerous other metrics could potentially be computed for each individual, including RT distribution factors.

In the processing of information by the brain, there are “bottom-up” sensory processes and “top-down” cognitive processes. Sensory processes involve the sense organ (in this case, the eye) and the direct pathway to the primary cortical brain region (in this case, the occipital cortex). There are also numerous pathways through the brainstem to activate the brain in response to an incoming stimulus. However, there are also higher-level cortical processes (in this case involving the frontal and temporal lobes) that contain the knowledge acquired by the person performing the task and can direct the processing of the incoming information. These “top-down” cognitive processes, including “efferent control” (Pribram, 1967), are responsible for guiding the neural processing during these tasks (Stanislaw and Todorov, 1999). Signal detection theory suggests that mathematical relationships between responses toward stimuli presented during a task explain the effects that experimental manipulation and clinical phenomena have on task performance, but this theoretical approach may not apply to a CRT that is constantly providing new information.

### Neural processing – the effects of aging

An important question is the extent to which processes change over the lifespan and are affected by disorders such as dementia. Age alters the ability to behave as instructed and affects information processing operations which store and search for and retrieve factual and declarative information previously consolidated in EM (Baddeley et al., 2019). Age (including age-related neurodegenerative processes) is also well known to affect the ability to learn and expend effort during tests (Luo and Craik, 2008). These effects are greater on some tasks than on others (Grady and Craik, 2000). For instance, slight age-related decrements occur during more automatic and stimulus driven tasks that require minimal instruction. Examples of cognitive tests that are less affected by age include STM tasks (repeating items over brief spans or without distraction), recognition memory tasks assessing remote LTM, accessing distant information, and WM tasks requiring the person to hold a sequence of items for a few seconds before repeating the string.

In the original AD description (Alzheimer, 1907), the patient could repeat words but not recall them after distraction. Similarly, the progressive effects of age are more disruptive in EM tasks that increase immediate involvement, such as instructing the user to hold or manipulate material in memory beyond the attention span or to process additional information while maintaining the first set of material in memory. These age effects are substantial during tasks that instruct the user to initiate processing, as is done during free or cued recall, to manipulate information held in memory, or to maintain information while concurrently processing incoming stimuli (Luo and Craik, 2008). As the stimuli become progressively more complex, the analytical requirements increase, and the inadequacies of the information processing system become more apparent, thus reflecting the continuum of normal cognitive function progressing to mild cognitive impairment and memory dysfunction (Hall et al., 2015; Ashford, 2023).

The main issue addressed here is to what extent the metrics of a CRT can provide information about the specific function of EM processes along the continuum of age. Age may alter instruction-directed explicit, intentional, and self-directed control of attention and learning during memory monitoring, encoding, the transition of declarative information to concept, and the accessing of cognitive processes. While age alters the ability of stimuli to affect processing, it does so without altering non-declarative implicit procedural memory involving automatic engagement of these events.

Consistent with the observations that age alters storage in and retrieval from EM differently than it alters the temporary maintenance of that information in STM, it is expected that analysis of the CRT will show specific age-related changes. Thus, inability to intentionally self-direct control of processes will affect the encoding of a stimulus the first time it appears and limit the depth of encoding. This test of EM may specifically reflect the common complaint by older adults about their difficulty in recalling information from semantic and episodic categories in LTM, as well as slowed processing and recognition. These effects on attention, learning, memory, and cognition begin in early life, even before 20 years of age, but the impact is usually extremely small and not problematic until after 60 years, at which time exponential decline becomes noticeable. However, variation in performance on such tasks also occurs and becomes more substantial across individuals with increased age (Ashford and Bayley, 2013; Malzbender et al., 2024).

### Hypotheses

The effects of age on the ability of the user to engage elaborative encoding strategies that enable transfer of information into EM storage and the ability to execute processes as instructed (Jonides et al., 2000), though central in AD and related dementias, are difficult to measure. Traditionally, these processes are measured using the face-to-face administration practices of neuropsychological assessment tools that involve trained professionals. Computer implementations of such tasks still do not provide the depth of processing provided with assessment by trained professionals. Importantly, most computer-based tools are not focused on EM and do not provide appropriate or precise measures of EM essential for clinical evaluation or follow-up or research outcomes (Sabbagh et al., 2020; Chan et al., 2021; Ashford et al., 2022b; Ding et al., 2022; Ashford, 2023; Chithiramohan et al., 2024).

MemTrax provides a method for quickly and precisely measuring EM, the central process affected by AD and several related dementias. This study re-examined behavioral data produced by the online application of the MemTrax CRT (Ashford et al., 2019), using an approach that separates TC into HITs and CRs, and a novel analytic method for response time, reverse exponential (RevEx) (Ashford et al., 2022a). These variables were further examined in this study to determine relationships with age and sex.

The hypotheses are as follows:

1. First we hypothesized that distributions for HITs and CRs are different phenomena, so the TC value is actually made-up of unrelated variables and does not represent a single function.
2. Secondly, we hypothesized that HITs and CRs follow separate probabilistic patterns, and potentially have different clinical utility.
3. The third hypothesis was that RT-HITs correspond to HITs, less to TC and not to CRs (or FAs).
4. We also hypothesized that the temporal distribution of RTs for both HITs and FAs follow the RevEx model.
5. And lastly, we hypothesized that these metrics would decline with age but would not differ between the sexes.

This analytical approach is novel and provides value and practical insight to the function of EM, which could be related to clinical disorders.

## Materials and methods

### Development of the MemTrax CRT

The first publication of the MemTrax CRT was based on audience presentations using a PowerPoint slide display and paper response forms administered to 1,018 subjects between July 2007 and June 2008 at 25 community sites (Ashford et al., 2011b).

HAPPYneuron, a French company, provided the first major online application of MemTrax between September 22, 2011 and August 22, 2013, with over 30,000 tests taken.^2^ Users were asked to sign up and provide year of birth, month of birth, sex, level of education, though there was no method for verification of this information. The first analysis of the HAPPYneuron data focused on the effects of age and sex on TC (as percent correct) and mean response time (RT) for 18,007 individuals taking the test for the first time (Ashford et al., 2019). There was a small, but significant adverse effect of age on TC and RT-HITs, and TC decreased as RT-HITs increased. While more than 4 times as many women took the test as men, there were no significant performance decrements with age that differed between the sexes.

In the next implementation of MemTrax, May 27, 2014 to May 7, 2022), 602,272 individuals took the test, and there were 344,165 first-time users identified. This analysis divided TC into HITs and CRs (Ashford et al., 2022a). The number of HITs corresponded significantly with faster RT-HITs, though CRs did not. In fact, those with the fewest CRs (most FAs) had the fastest RT-HITs. The distribution of RTs to HITs was basically the same in both studies, showing a positively skewed distribution typical of RT studies. However, examination of the cumulative RT curve in this study showed that the distribution fit a negative survival curve with a 2-factor exponential equation, characterized by an *R*^2^ exceeding −0.99, thus explaining more than 99% of the variance (RevEx model).

### Initial population selection

The present study is a reanalysis of the HAPPYneuron data. The full HAPPYneuron data set included 30,435 times the test was taken online. Of these times, 25,146 unique users were identified. In this set, 1,483 individuals noted an age over 100 years (date of test – provided birthdate, month and year only, mostly birthdays not entered correctly). Review and approval of the protocol was provided by the Stanford Institutional Review Board and authorization for anonymous data collection and analysis was only granted for individuals over the age of 21 years, so, 9,794 tests for individuals who indicated age was less than 21 years were eliminated, leaving 20,641 individuals. Further, 1,372 individuals did not indicate a birthdate, leaving 19,269. Of these, 89 duplicate IDs were found and removed, leaving 18,979 unique individuals aged 21–100 years who took the test for the first time.

### Study design

**FIGURE 1:**
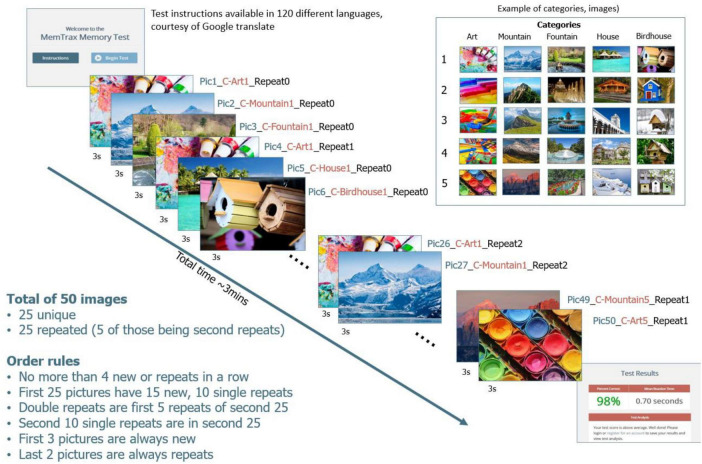
Graphical display of the MemTrax CRT.

The online HAPPYneuron MemTrax task presented 50 images, 25 new and 25 repeat images, in a pseudo random order (Figure 1), with the instruction to respond to repeated images as quickly as possible (Ashford et al., 2019). HAPPYneuron changed the pictures each month and used one of four order sets. The required response was a spacebar tap. Users were allowed up to 3 s after stimulus presentation to respond. The 3-s time was acceptable for individuals of all ages. When an individual made a response, the next image was shown immediately (or at least after 50 ms).

The stimuli were complex, “interesting” pictures from five separate categories, with five pictures from each category. Of the five pictures, four were repeated once and one repeated twice. The first 2 pictures were always new, but there could have been up to 4 new or old pictures in a row, with the interval between the first show and the repeat varying from 1 to 45 pictures. RT to each stimulus was recorded and available for analysis. A HIT was defined as a response to a repeated stimulus that occurred between 50 and 2,900 ms after the stimulus onset, and the RT-HIT was recorded. CR for an initial presentation occurred when no response was made after a stimulus onset for 3,000 ms. A MISS was identified when there was no response to a repeated stimulus between 50 and 2,900 ms. A FA was any response to an initial presentation of a stimulus, and the RT-FA was recorded. The distribution and time of responses of HITs represents EM function.

### Sample selection

Of the first tests taken, about 2.2% of the full sample (561) made no responses (0/50) and an additional 0.7% had no better than random chance TC responses (30/50, 60% correct, ranging from 5 HITs with 25 Correct Rejections to 25 HITs with 5 Correct Rejections). Among this group, 629 tests had less than 60% correct, many with no responses, leaving 18,350 individuals with better than chance performance. Also, 238 (1%) had fewer than 30 (60%) TCs. There were 64 individuals who had 30, 31, or 32 TC (mostly due to missed true positive responses, few responses). Users with 32 or fewer TC (<65%) (better than 60%, which would still be better than chance by 2 additional correct responses) were removed (permissible range: 8/25 HITs with 25/25 CRs to 25/25 HITs with 8/25 CRs). Of these remaining users, 494 had fewer than 16 HITs and 126 had fewer than 16 CRs.

There were 61 users with RT-HITs greater than 1,800 ms, and 599 with less than 550 ms. There were 38 users with RT-HITs between 266 and 538 ms, but none of these individuals had more than 30/50 total correct (one with 540 ms had 49/50 correct, next fastest 558 ms had 50/50 correct). Thirteen users had average RT-HITs slower than 2,000 ms (max 2,376) and were omitted from the analysis.

**FIGURE 2:**
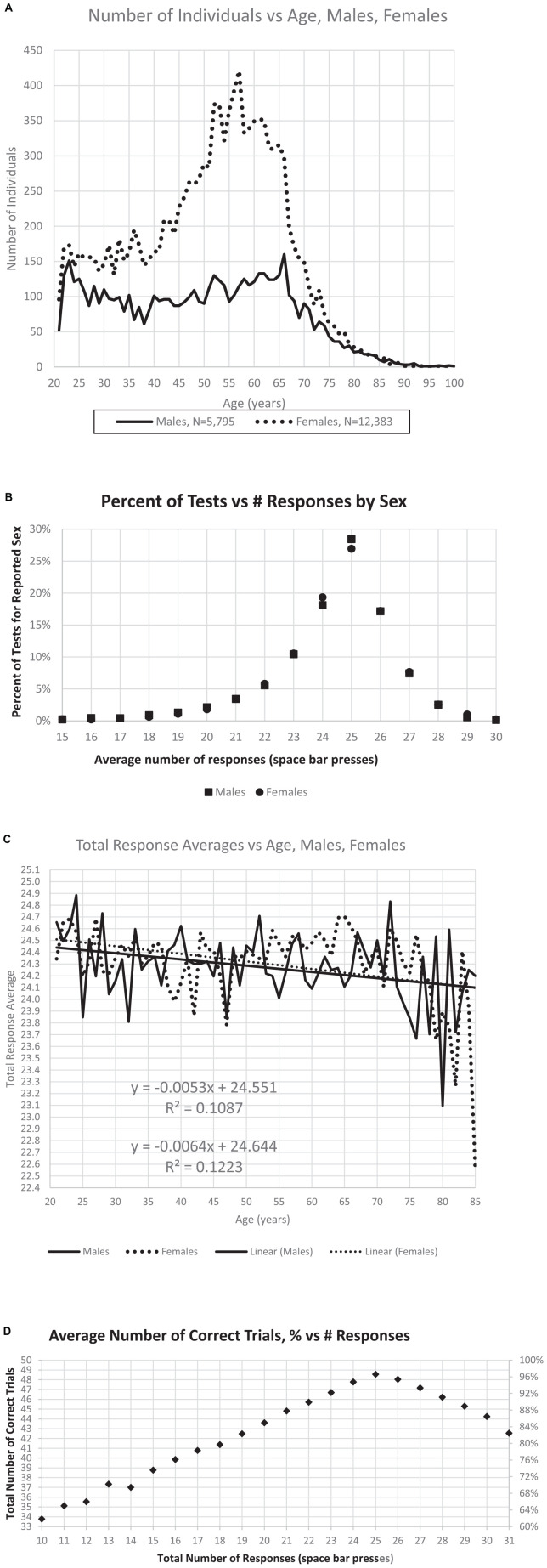
Relationships between number of individuals, sex, age, and responses. **(A)** Distribution of the number of male and female users by age. **(B)** Percent of responses per test is maximum at the ideal number, 25, with little difference related to sex either at this maximum, or with fewer responses or more responses. **(C)** There is a slight decrease of total responses with age, though the effect of sex with respect to age is minimal. **(D)** Maximum correct occurs at 25 responses, with a relatively symmetric decline of percent of correct trials increasing to 30 responses or decreasing to 20 responses.

After this selection process, the sample analyzed here included 18,265 individuals. In this group, 5,795 men, 12,383 women (Figure 2A), 87 sex not indicated. There was little difference in the average number of responses for males and females (Figure 2B), the optimal number being 25. The average number of responses declined slightly with age (Figure 2C). The selected individuals had performances of 33–50 TC with RT-HITs 540–2,000 ms. Of these users, 364 (1.5%) had fewer than 35 TC (70% correct trials), and 774 (3%) had fewer than 40 TC (80% correct trials) (Figure 2D). In examining the incorrect responses to images shown for the first time, there were 7,201 individuals with average RT-FAs less than 558 ms.

### Statistical analysis

**FIGURE 3:**
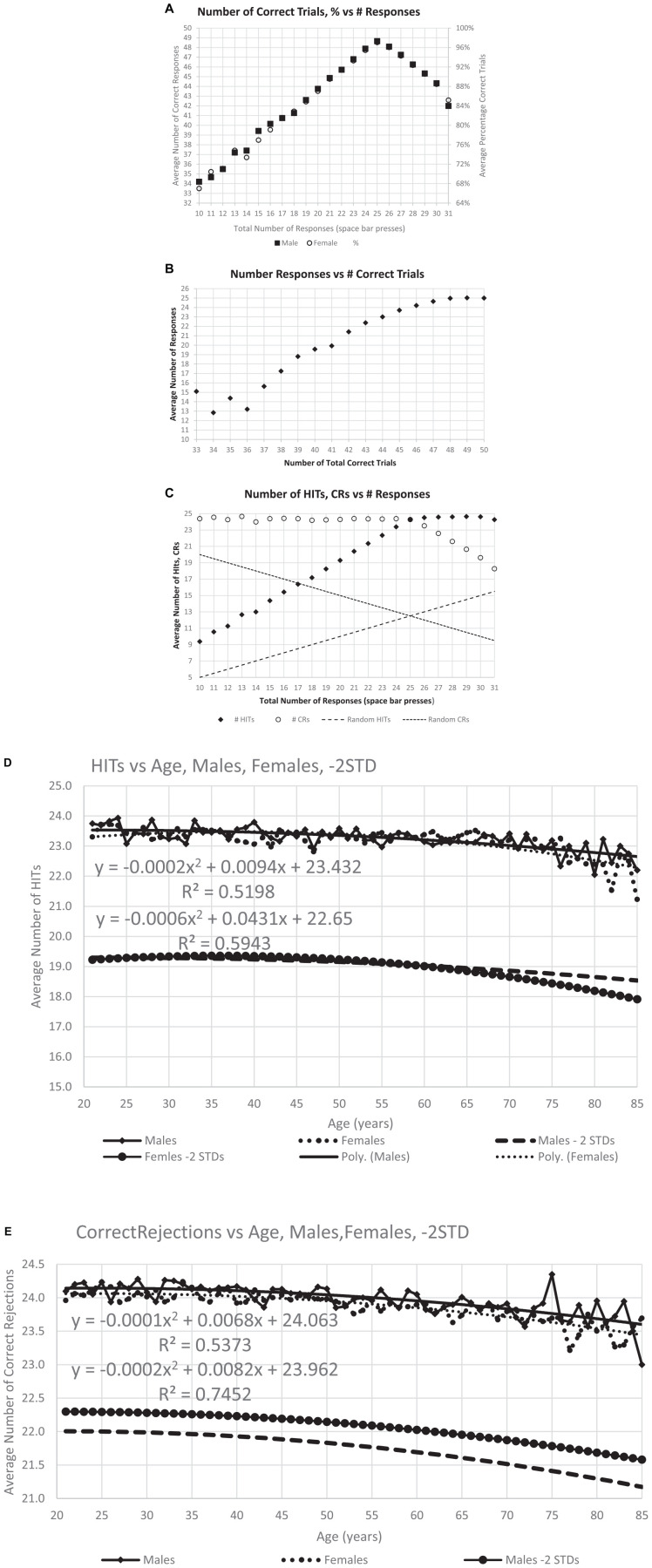
Correctness of responses. **(A)** The maximum number of average correct response, 48.6, occurs at 25, the optimal number of responses, and the distribution is similar for men and women. **(B)** Same data with average number of responses for each level of total correct responses for individuals at that level, particularly showing the decreased number of responses with fewer total correct. **(C)** This analysis breaks the total correct into HITs and CRs and shows the number of HITS and CRs for each specific response count. Dashed lines show the calculated numbers for random performance, indicating the degree to which the responses of individuals in general are not random. Panels **(D,E)** show the average number of HITs and CRs averaged for each age, the quadratic (polynomial) regression line, and a line 2 SD for the whole population below the regression line.

**FIGURE 4:**
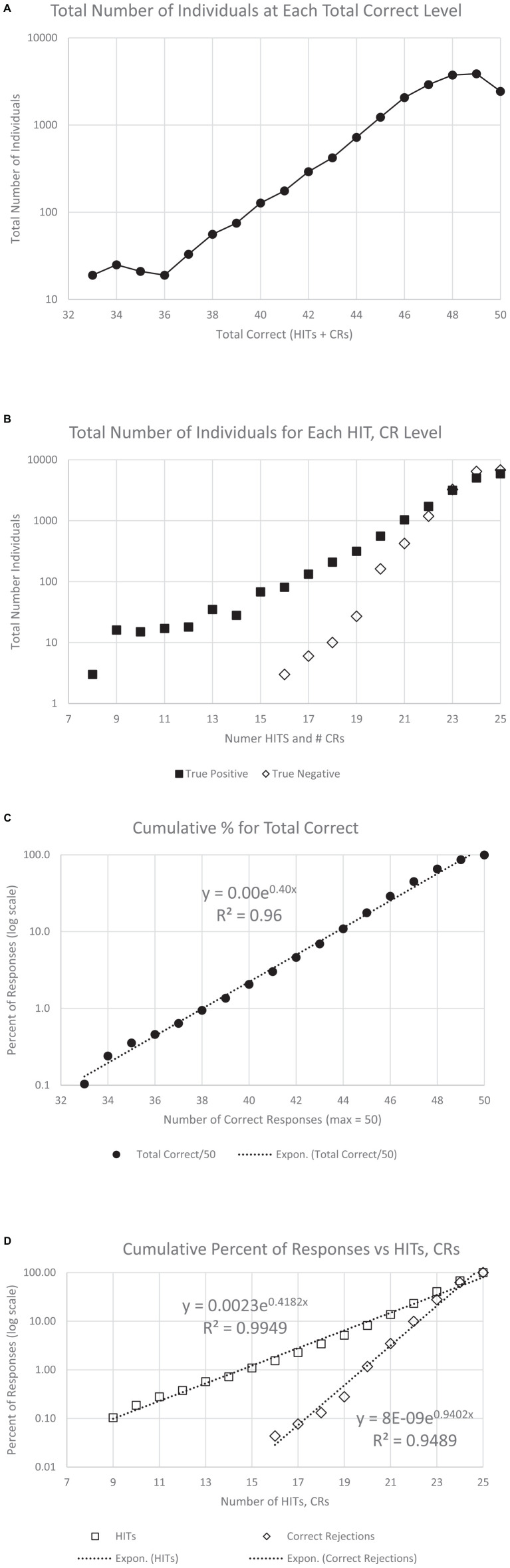
Number of users and cumulative percent of individuals associated with each number correct. **(A)** Number of users for total correct. Panel **(B)** shows the same data separated by HITs and CRs. The next graphs show the cumulative percent for the population: panel **(C)** for TC (total correct); and panel **(D)** for HITs and CRs. Note that all three cumulative plots show a major exponential relationship between responses and correctness, with the highest relationship shown with HITs, *R*^2^ = 0.995. The numerical values of the raw data are shown in Table 1A for clinical purposes.

**FIGURE 5:**
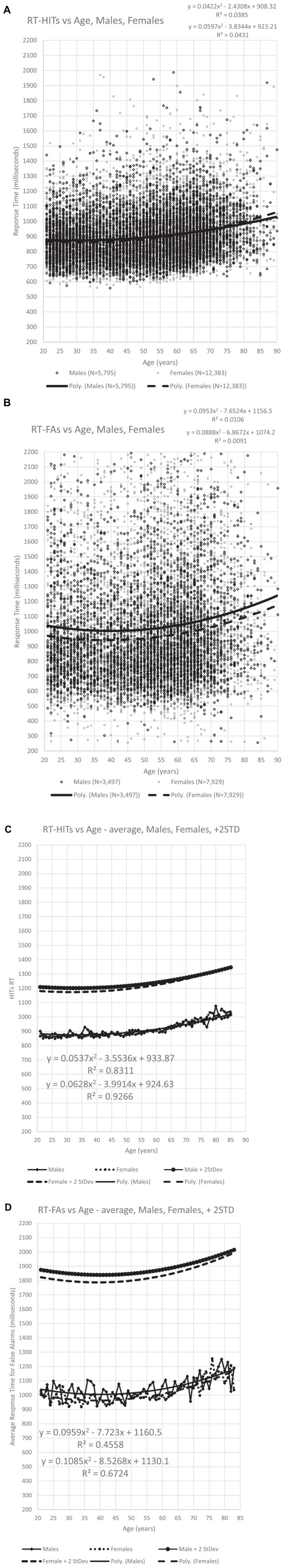
The average RTs performed by all participants with a polynomial regression and *R*^2^. Panel **(A)** shows the RT averages for individuals’ HITs, showing the slight increase of RT-HITs with age, similar for men and women, as previously reported (Ashford et al., 2019). Panel **(B)** shows the RT averages for FAs, with fewer individuals, both men and women. A small difference in men and women is present, with a slightly higher proportion of men having FAs and slightly slower RT-FAs. Panel **(C)** shows the average for each age, providing a similar pattern of RT change with respect to age as the regression line in panel **(A)**, but the significance with respect to age is greatly increased. Panel **(D)** shows the same for FAs, also comparable to the raw data, but the significance of the regression lines is less than for the RT-HITs.

**FIGURE 6:**
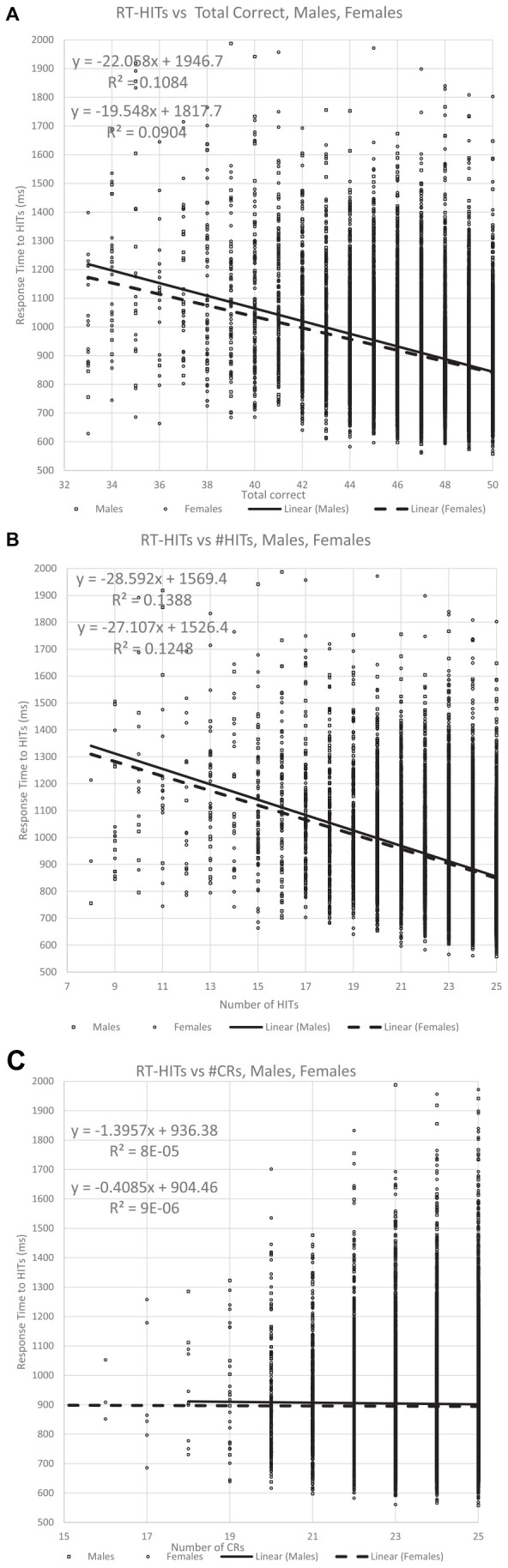
The relationship of the RTs for Hits for all participants with respect to correctness. Panel **(A)** shows the relationship with TC (total correct) responses, while panel **(B)** shows the portion of correct responses contributed by HITs, which is more significantly related to the RT for the HITs than for TC. By contrast, panel **(C)** shows the portion of correct responses contributed by CRs, and this metric has essentially no relationship to the RT for HITS.

**FIGURE 7:**
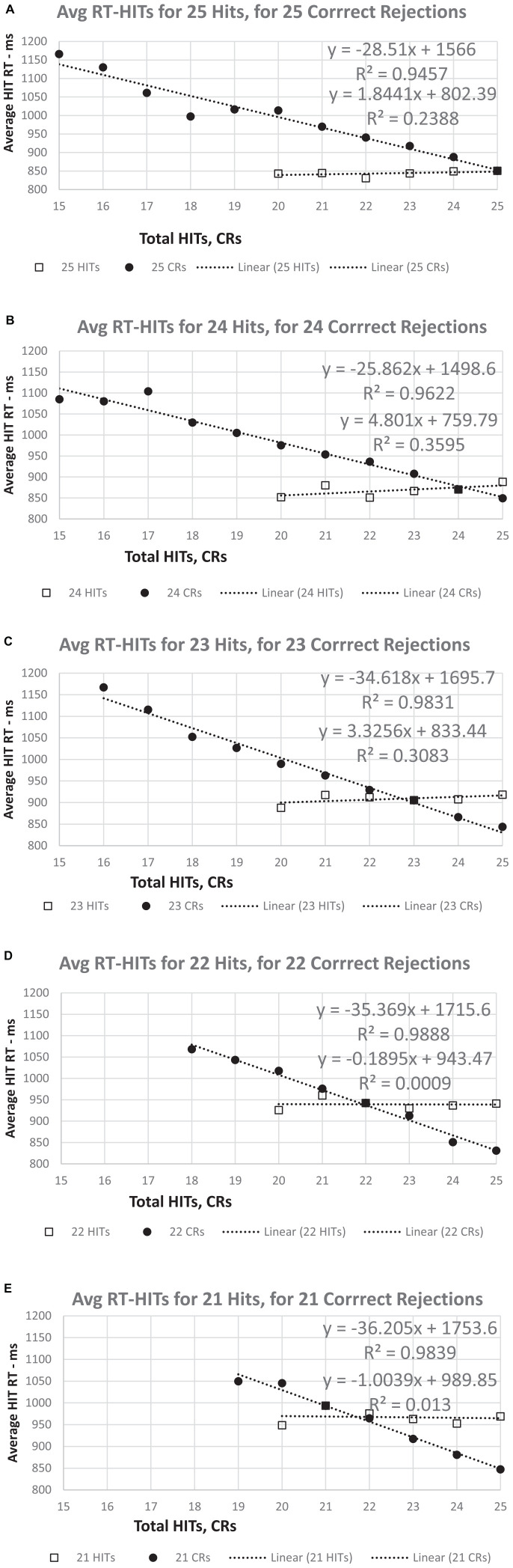
**(A–E)** The RT for HITs apportioned by number correct for HITs and CRs. For a set number of HITs (25 to 21), there is essentially no relationship between RT-HITS and the number of CRs. However, regardless of the number of CRs (25 to 21), the RT-HITs have a strong linear relationship between the RT-HITs and the decreasing number of HITs. Note that for 25 CRs, the value is the same in all five graphs, though the slope of the relationship between RT-HITs and number of HITs declines slightly as the number of CRs decreases. By contrast, the slight positive relationship between CRs and RT (more CRs, fewer FAs, slower RT-HITs) shows a progressive decrease overall across the graphs.

**FIGURE 8:**
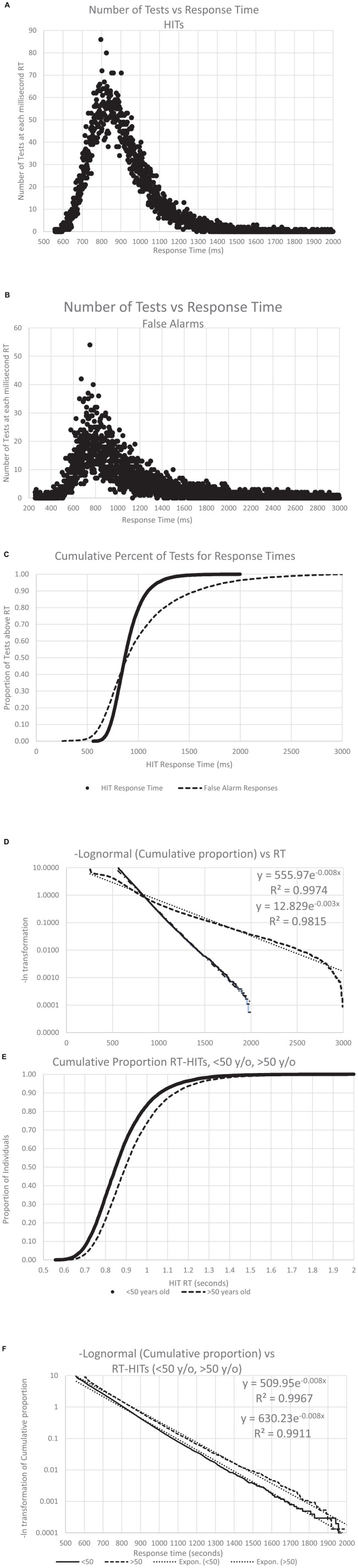
The number of individuals with an RT, both for HITS and FAs, across all the analyzed data. Panel **(A)** shows the distribution of the RT-HITs, by millisecond, the number of RT-HITs across the user population for each millisecond, with a range from 550 to 2,000 ms. Panel **(B)** shows the distribution of the RT-FAs, with a range from 250 to 2,999 ms. Note the skewed distribution of both plots. Panel **(C)** shows the curves for cumulative distribution for both RT-HITs and RT-FAs, clearly showing the different distributions, with 45% of the individuals having faster RT-FAs than RT-HITs, while there are 55% of the users for whom RT-HITs were faster than RT-FAs, with a notably larger number substantially slower. Of course, there were many more responses for HITS across users than FAs, but that discrepancy would not be expected to affect this distribution. **(8)** Taking the negative natural log of each slope, there is again a very strong reverse exponential relationship (RevEx), explaining well better than 99% of the variance of RT-HITs and only slightly less for RT-FAs. Panel **(E)** shows the cumulative distribution of RT-HITs for those below and above the median age of 50 years old, with the older group being slightly slower across all times than those under 50. **(F)** The negative natural logs again explain more than 99% of the variance, but the slope shows that RT-HITs are slightly longer for those over 50, suggesting that the difference in those at the slower end are progressively even slower with increased age.

To test the hypotheses, we determined HITs, CRs, FAs, TC, and RT-HITs and RT-FAs for each age group and sex. The following plots correspond to the five hypotheses:

1. First hypothesis, distributions for TC, HITs, and CRs (Figures 3A–C).
2. Second hypothesis HITs, CRs probabilistic patterns (Figures 4A–D).
3. Third hypothesis, RT-HITs corresponds to HITs (Figures 6A–C, 7A–E).
4. Fourth hypothesis, RTs follow the RevEx model (Figures 8A–D).
5. Fifth hypothesis: metrics decline with age not differing by sex (Figures 2C, 3D, E, 5A–D, 8E, F).

## Results

### Analysis of data from selected population

The age distribution shown in Figure 2A was nearly identical to that of the prior study of this data set (Ashford et al., 2019) with similar numbers of users, 5,795 male and 12,383 female (87 not declared), and more than twice as many female users as male, with the major group being women between 40 and 70 years of age (Figure 2A). Further, the pattern of responses follows the pattern of descriptions of a much larger data set, which had no user information, e.g., age, sex, and education (Ashford et al., 2022a). The distribution of responses is also similar, showing 28.5% of the male users and 26.9% of the female users making exactly 25 responses (the ideal number), as shown in Figure 2B. There was about a half point difference in the average number of responses between ages 21 (24.5) and 75 (24.1) years (Figure 2C). The average number of responses was 24.30 for men and 24.39 for women. The regression lines for age for men and for women were similar, decreasing from 24.5 at age 21 years to 24.1 at age 85 years (Figure 2C). Users making exactly 25 responses averaged 97% correct, while those making only 20 responses (5 too few) averaged 87% correct and those making 30 responses (5 too many) averaged 89% correct (Figure 2D). These results are essentially the same as the data set in a recent analysis of fully anonymous data without demographic information from www.memtrax.com (Ashford et al., 2022a).

### Correctness of responses

The average number of responses per participant was compared to the average number of correct responses. The modal correct responses occurred at 25 (Figure 3A), the ideal number (Figure 2B). A steeper decline in correctness occurred for fewer than more responses. Further, the average number of responses also declined as the total number of correct responses decreased (Figure 3B). There was a marked proportional relationship between number of responses and HITs below 25 responses, and a similarly marked but inversely proportional and unrelated relationship for CRs above 25 responses (Figure 3C). However, there was a relatively small effect of age on number of HITs or CRs (Figures 3D, E).

### Population distribution of correct responses

There was a progressive increase in the total number of users who performed from 66% correct (33/50) to 96% correct (48/50) (Figure 4A and Table 1A, TC). Based on the relationship between HITs and CRs, there was a substantially steeper slope for CRs than HITs (Figure 4B and Table 1A, HITs and CRs). Cumulative percentiles of these data (Figures 4C, D) showed a clear progression that explains over 96% of the variance for total correct, 99.5% for the HITs, and 95% for the CRs. Clearly, HITs are more reflective of a different dimension of correctness than CRs. These distributions of correctness (Ashford et al., 2022a), provide a performance-based estimation of a person’s cognitive ability (data suitable for clinical use in Tables 1A, B).

**TABLE 1A:** Total correct (TC), total HITS, and total CRs, with percentiles and *Z*-scores relative to the mean numbers for each measure.

	Percentile	*Z*-score
**TC**		
50	99.90	3.09
49	86.65	1.11
48	65.41	0.40
47	44.87	−0.13
46	28.94	−0.56
45	17.63	−0.93
44	10.90	−1.23
43	6.93	−1.48
42	4.62	−1.68
41	3.02	−1.88
40	2.06	−2.04
39	1.36	−2.21
38	0.95	−2.35
37	0.64	−2.49
36	0.46	−2.60
35	0.36	−2.69
34	0.24	−2.82
33	0.10	−3.08
**HITS**		
25	99.90	3.09
24	67.91	0.47
23	40.51	−0.24
22	23.21	−0.73
21	13.82	−1.09
20	8.18	−1.39
19	5.14	−1.63
18	3.41	−1.82
17	2.27	−2.00
16	1.54	−2.16
15	1.09	−2.29
14	0.72	−2.45
13	0.57	−2.53
12	0.38	−2.67
11	0.28	−2.77
10	0.19	−2.90
9	0.10	−3.08
**CRs**		
25	99.90	3.09
24	62.83	0.33
23	27.71	−0.59
22	9.96	−1.28
21	3.48	−1.81
20	1.17	−2.27
19	0.28	−2.77
18	0.13	−3.01
17	0.08	−3.17
16	0.04	−3.33

**TABLE 1B:** Average number of HITs and CRs and −1, −1.5, and −2 SD, by 10-year age cohorts.

	Mean	−1 SD	+1.5 SD	−2 SD
**HITS**				
**Male age**				
21–31	23.5	21.4	20.4	19.3
31–40	23.5	21.4	20.4	19.3
41–51	23.4	21.3	20.3	19.2
51–60	23.3	21.2	20.2	19.1
61–70	23.2	21.1	20.1	18.9
71–81	23	20.9	19.9	18.7
81–90	22.8	20.6	19.5	18.5
**Female age**				
21–31	23.4	21.3	20.3	19.3
31–40	23.4	21.4	20.4	19.4
41–51	23.4	21.3	20.3	19.3
51–60	23.2	21.2	20.2	19.1
61–70	22.9	20.9	19.9	18.8
71–81	22.5	20.4	19.4	18.4
81–90	21.9	19.9	18.9	17.9
**CRs (correct rejections)**				
**Male age**				
21–31	24.1	23.2	22.8	22.3
31–40	24.1	23.2	22.8	22.3
41–51	24	23.1	22.7	22.2
51–60	23.9	23	22.6	22.1
61–70	23.8	22.9	22.5	21.9
71–81	23.6	22.7	22.3	21.8
81–90	23.4	22.5	22.1	21.6
**Female age**				
21–31	24	23	22.5	22
31–40	24	23	22.5	22
41–51	23.9	22.9	22.4	21.9
51–60	23.8	22.8	22.3	21.8
61–70	23.6	22.6	22.1	21.6
71–81	23.4	22.4	21.9	21.4
81–90	23.2	22.2	21.7	21.2

More than 2 SD is generally considered abnormal. Two values outside 1.5 SD are considered possible impairment.

### Response times to HITs and false alarms

The mean RT-HITs and the mean RT-FAs were calculated for each individual. The fastest RT-HITs mean was 538 ms. There were 13 users removed from this analysis for RT-HITs slower than 2,000 ms, which would be more than 3 SD beyond the mean. Among males, 2,298 (39.7%) made no FAs (range of non-zero FAs: 256–2,993 ms), while among females, 4,454 (36.0%) made no FAs (range of non-zero FAs: 256–2,286 ms).

Individual variability was found to be a substantially greater contributor to variance in RT than age. The plots and regression lines for RT-HITs show tremendous variability. However, there was essentially no difference in the distribution with respect to age for men and women for RT-HITs (Figure 5A). Since many individuals had no FAs, the analysis of the RT-FAs metric is problematic and shows more variability. The RT-FAs show that women were slightly faster than the men across the age range in their RTs for FAs (Figure 5B).

The relationship between participant scores averaged for each year of age with calculated SD shows a substantial relationship with age, which is pronounced for the RT-HITs (Figure 5C). In spite of the significance of this effect, the magnitude is less than 15% from 20 to 85 years (about 880–1,000 ms) and small compared to the population variability (data suitable for clinical use in Table 1C). RT-FAs are overall slower than RT-HITs and show even more variability (Figure 5D). There is essentially no relationship between RT-FAs and RT-HITs (males: *y* = 0.58*x* + 101, *R*^2^ = 0.025; females: *y* = 0.64*x* + 67, *R*^2^ = 0.29).

**TABLE 1C:** Response times for HITs in milliseconds, with percentiles and *Z*-scores relative to the mean for each 10 ms time bin.

RT-HITs	Percentile	*Z*-score	RT-HITs	Percentile	*Z*-score
560	100.0	3.9	1,100	9.4	−1.3
580	100.0	3.4	1,120	8.1	−1.4
600	99.9	3.0	1,140	7.1	−1.5
620	99.6	2.6	1,160	6.1	−1.5
640	99.1	2.4	1,180	5.2	−1.6
660	98.2	2.1	1,200	4.5	−1.7
680	96.7	1.8	1,220	4.0	−1.8
700	94.5	1.6	1,240	3.4	−1.8
720	91.1	1.3	1,260	2.9	−1.9
740	87.4	1.1	1,280	2.4	−2.0
760	82.6	0.9	1,300	2.1	−2.0
780	77.1	0.7	1,320	1.8	−2.1
800	70.9	0.6	1,340	1.6	−2.1
820	64.7	0.4	1,360	1.4	−2.2
840	58.6	0.2	1,380	1.2	−2.3
860	52.3	0.1	1,400	1.0	−2.3
880	46.3	−0.1	1,420	0.9	−2.4
900	40.7	−0.2	1,440	0.8	−2.4
920	35.6	−0.4	1,460	0.7	−2.5
940	31.0	−0.5	1,480	0.6	−2.5
960	26.9	−0.6	1,500	0.6	−2.5
980	23.4	−0.7	1,520	0.5	−2.6
1,000	20.3	−0.8	1,540	0.5	−2.6
1,020	17.3	−0.9	1,560	0.4	−2.7
1,040	14.8	−1.0	1,580	0.3	−2.7
1,060	12.7	−1.1	1,600	0.3	−2.8
1,080	11.0	−1.2			

### Response time relationship to response correctness

This analysis first plotted RTs as a function of TC (total correct = HITs + CRs) and then that value was separated by HITs and CRs. As has been shown in prior studies, the RT-HITs have an inversely proportional dependence on total correct responses with an *R*^2^ of about 10% (Figure 6A). However, there was a stronger relationship between RT-HITs and number of HITs, with an *R*^2^ closer to 13%, similar for men and women (Figure 6B). In contrast, the number of CRs had essentially no relationship with RT-HITs (Figure 6C). Therefore, the number of HITs was the factor generating the relationship between correctness and RT-HITs, and the number of CRs does not contribute to this relationship.

Additional plots that compared the RT-HITs in association with the numbers of HITs and CR for users scoring between 21 and 25 (perfect) correct for each variable were constructed to examine the dichotomy of HITs and CRs (Figures 7A–E). When the number of CRs is held constant, there was a significant and linear relationship with the number of HITs, with a higher proportion of HITs associated with faster RT-HITs. However, when the number of HITs was held constant, there was a minimal relationship of the RT-HITs with the number of CRs, actually starting out slightly negative (suggesting a few CRs could make RT faster), but with lower HIT counts. These graphs clearly show there is no relationship between RT-HITs and CRs.

### Response time distributions, the RevEx model

As shown previously with a different data set (Ashford et al., 2022a), RT-HITs in this CRT showed a positively skewed distribution (Figure 8A). Also, the present analysis of the broader distribution of RT-FAs showed a similar positively skewed distribution (for the reduced number of individuals making FAs; Figure 8B). When the two curves were transformed with a cumulative distribution, the RT-HITs showed a tighter distribution, and the RT-FAs distribution showed a faster RT about 40% of the time and a progressively slower RT for 60% of the users (Figure 8C). When a negative logarithm of the cumulative distributions was plotted, a reverse exponential regression line (RevEx) explained more than 99% of the variance for the distribution of the RT-HITs. There was a substantially different distribution for RT-FAs, though the exponential regression line still explained better than 98% of the distribution. For both curves, there was a notable fall-off at the longer RTs suggesting that the rare extremely slow responses are related to phenomena that are not part of the test activities.

### Response time distribution and age

To assess the effect of age on the RT-HITs distribution, individuals were divided according to the median age, 50 years old, and the RevEx curves were calculated for the younger and older groups. The users under 50 years old had a slightly faster distribution of RT-HITs than those over 50 years old (Figure 8E). Of further interest, the exponential regression function of the cumulative distribution showed that the users with the slower RTs had greater age-related slowing (Figure 8F). The statistical and age-cohort distributions are provided (Tables 1C, D), though there is a basic question of whether the definition of abnormality can be made statistically or in relation to the patient’s age or that of a “young” and “healthy” adult.

**TABLE 1D:** Response times for HITs and FAs and +1, +1.5, and +2 SD, in milliseconds, by 10-year age cohorts.

	Mean	+1 SD	+1.5 SD	+2 SD
**RTs for HITS**				
**Male age**				
21–31	879	1,042	1,124	1,205
31–40	876	1,039	1,121	1,202
41–51	884	1,047	1,129	1,210
51–60	902	1,066	1,148	1,229
61–70	932	1,095	1,177	1,258
71–81	972	1,135	1,217	1,298
81–90	1,023	1,186	1,268	1,349
**Female age**				
21–31	864	1,020	1,098	1,176
31–40	863	1,019	1,097	1,175
41–51	874	1,030	1,108	1,186
51–60	897	1,053	1,131	1,209
61–70	933	1,089	1,167	1,245
71–81	982	1,138	1,216	1,294
81–90	1,043	1,199	1,277	1,355
**RTs for FAs (false alarms)**				
**Male age**				
21–31	1,027	1,444	1,653	1,861
31–40	1,008	1,425	1,634	1,842
41–51	1,008	1,426	1,635	1,843
51–60	1,028	1,445	1,654	1,862
61–70	1,067	1,484	1,693	1,901
71–81	1,125	1,542	1,751	1,959
81–90	1,202	1,619	1,828	2,036
**Female age**				
21–31	984	1,396	1,602	1,809
31–40	965	1,377	1,583	1,789
41–51	968	1,380	1,586	1,792
51–60	992	1,404	1,610	1,816
61–70	1,038	1,450	1,656	1,862
71–81	1,106	1,518	1,724	1,930
81–90	1,195	1,607	1,813	2,020

More than 2 SD is generally considered abnormal. Two values outside 1.5 SD are considered possible impairment.

## Discussion

Standard clinical tests of neuropsychological and cognitive function utilize face-to-face, paper-and-pencil tests administered by trained neuropsychologists or technicians. However, new computerized tests may be able to provide greater precision and improved efficiency. In particular, the tests used for measuring learning, memory, and cognitive functions in participants, often self-reporting concern, are a major problem in clinical settings. Direct face-to-face interactions may bias observations and not provide adequately precise assessment of these critical brain functions. Further, such data are susceptible to inter- and intra-rater variability that contributes to type 1 and 2 error when included in the diagnostic process. The present study examined whether behavioral metrics reflecting learning, memory and cognition provided by participants 21–100 years old who voluntarily performed the MemTrax computerized application of a CRT could yield clinically meaningful data.

This easy-to-use MemTrax test was accessed by online users without known clinical concerns or cognitive impairment and without supervision. Participants in the present dataset provided no clinical information or indications of supervision. Though there were self-report questions, they could not be verified. Participants may have had different motivations for accessing the task. For example, the preponderance of participants were women in the early menopause and menopause range (40–65 years old), which is known to be associated with memory complaints (such as “brain fog”); though, after 80, when women outnumber men 2:1 in the general population, there was an equal number of users across both sexes. Comparing male and female users across the age range and establishing the continuity and the validity of the data produced by MemTrax is substantiated and replicates prior studies (Ashford et al., 2022a).

MemTrax is in the family of SDTs which uses the CRT paradigm. However, there is an important question regarding whether Signal Detection Theory is applicable to this CRT. The stimuli presented by the MemTrax CRT activate the range of sensory and cognitive processes that IPMs suggest are occurring in the brain of a participant attending to and responding as instructed to those stimuli during the test. Signal Detection Theory provides a mathematical framework to differentiate between target stimuli and non-target stimuli. With this theory, an image being shown for the first time would be the non-target, while the repeated image would be the target. In this model, the occurrence of target and non-target stimuli would theoretically each be normally distributed along a line of probability. Signal Detection Theory provides a format to calculate a d’ factor which defines how far apart the probabilities are for the two distributions. Also, there is a beta factor which defines whether the observer is more or less likely to respond to a target or non-target, along the continuum. A question raised by this study was whether the Signal Detection Theory concept of a *d*′ factor could be related to HITs and FAs, and whether a beta factor could relate the tendency to under- or over-respond and explain performance. However, since the distributions of HITs and FAs had separate relationships to TC and RT, there is no probabilistic relationship between them, and accordingly, Signal Detection Theory does not appear to apply to the analysis of the MemTrax CRT. This finding is consistent with similar studies of CRTs (Fuchs et al., 1999). In a CRT, the HITs represent a complex recognition event, not just the distinguishing of a signal from a noise, and the FAs represent the tendency to lack inhibition in the complex decision to respond or not respond. Thus, the HITs are an indication of the brain’s ability to store stimulus information in EM and recognize it later, a function of the temporal lobe (Ashford et al., 1998; Sherwood et al., 2020), which is particularly impaired by damage to the hippocampus (Stark et al., 2002), a hallmark of AD. Alternatively, the tendency to over-respond (lack of inhibition) is a frontal lobe issue (Konorski, 1972), unrelated to recognition ability, which occurs in distributed and interactive cortical networks that encode information into EM (Fuster and Bressler, 2012; Sherwood et al., 2020).

This study examined whether behavioral metrics in 18,245 participants between 21 and 100 years old who completed a simple demographic questionnaire and the MemTrax CRT showed a distribution of performance metrics appropriate for the analysis of memory function and whether those metrics were related to sex and age. Most participants performed this task with zero to six FA or MISS errors, suggesting that the internal state of the participant was the principal factor being measured, and external factors not related to the task had minimal effects on the quality and distinctiveness of the information processing.

The two types of errors, MISSes (1-HITs) and FAs (1-CRs), had different effects on RT-HITs. In this case, incrementally increasing the number of FAs on trials with a set number of MISSes does not increase RT-HITs (Figures 6, 7). However, incrementally increasing the number of MISSes on trials with a set number of FAs progressively slowed RT-HITs. This dichotomy suggests that incorrect analyses of non-matching and of matching stimuli resulting in FAs and MISSes have different effects on correct analysis of matching stimuli associated with HITs. Therefore, incorrect analysis of novel non-matching stimuli resulting in FAs alters the effect that incorrect analysis of familiar matching stimuli resulting in MISSes has on RTs for correct analyses of matching stimuli producing HITs. The incorrect analysis of familiar matching stimuli, producing MISSes, is recognition failure. However, incorrect analysis of novel non-matching stimuli producing FAs has no slowing effect on RTs for correct analysis of matching stimuli producing HITs. Thus, the lack of familiarity of matching stimuli, which results in MISSes, indicates a disruption in the recognition process.

A recent MemTrax study (Ashford et al., 2022a) performed independent analyses of the HITs and CRs and showed a substantial difference in their distribution, and there was a closer relationship of RT-HITs to total HITS than to TC, while there was essentially no relationship between CRs and RT-HITs. These distributions reported here are essentially identical to those reported from the more recent MemTrax data set (Figure 3; Ashford et al., 2022a), providing strong reproducibility for MemTrax data across different settings.

Consistent with the study in large group settings (Ashford et al., 2011b), age appears to adversely affect performance on the MemTrax CRT. Presumably, age alters the sensitivity of the information processing apparatus and brain functions that encode then recognize an event, in this case, the visual processing system. Further, age is associated with decline in brain efficiency to expend effort to direct attention and initiate new learning that establishes familiarity and transition the information processing apparatus to a state of automaticity and habituation. As such, age degrades the capacity of STM to transfer information into EM to maintain representations. This deterioration of processing leads to slow and failing recognition responses and mediates the effect of MISSes on RTs for HITs, as seen in a study of similar parameters (Ratcliff et al., 2021). A separate process in which the individual is trying excessively hard to make responses results in FAs. Importantly, these different effects of errors on performance may alter mathematical relationships between these events, but differently than described by Signal Detection Theory (Stanislaw and Todorov, 1999). In the current study, an effect of age on impairing performance is clearly seen with HITS, CRs and RTs, though the age-effect is small with respect to inter-individual variability.

The effects of age on STM and EM and on error are not addressed by traditional SDTs that instruct individuals to attend to and detect occurrences of a specific “target” stimulus or stimuli, such as the Sternberg task (Sternberg, 1966). This traditional strategy produces data that illustrate a general proportional linear increase in RTs toward those stimuli in the elderly, with a disproportionate deterioration of those RTs related to memory function (Poon and Fozard, 1980; Bowles and Poon, 1982; Hines et al., 1982; Myerson et al., 1990; Yesavage et al., 1999; Ishihara et al., 2002). However, the utilization of a specific target has not been useful in producing data that describe a continuum identifying within- and between-group variation and a formula that distinguishes transition from normal to non-normal processing. This deficiency may reflect the effect of instruction on those operations in WM during the task. In these traditional SDTs, instruction sets a criterion that directs those operations in WM to deploy processes that maintain and compare the representation of the “target” in STM with the present stimulus. Thus, analysis resulting in a match or mismatch between that information in STM clears content from STM on that trial so that only the representation of the “target” occupies STM during the interval between presentations. The MemTrax CRT effect, pushing way beyond the immediate storage capacity of STM, alters the focus expended by operations in WM to deploy resources, so it is the neural processes of EM, predominantly related to medial temporal lobe function, which produce correct and incorrect responses. This difference is the critical factor provided by the CRT used in the present study. Accordingly, the MemTrax CRT is predominantly assessing the capacity, or decreased capacity, of EM, specifically the function of the medial temporal lobe.

In the MemTrax CRT, instruction to attend to all stimuli and detect repetition of a stimulus directs neural systems to deploy processes to maintain and compare each representation with those from other trials to detect a match. As such, each stimulus must be viewed first as new information in STM. The critical issue is whether the present stimulus matches information previously perceived. If there is no match, this information requires transfer into EM for use on subsequent trials (Baddeley and Hitch, 2000; Baddeley et al., 2019). The process of encoding the new information is presumably similar to neural functions associated with the P300 evoked potential, which is particularly disturbed in early AD, likely due to the critical underlying pathology affecting EM (Ashford et al., 2011a; Marizzoni et al., 2019). If the encoding is adequate, then the analysis of a repeated stimulus will result in a neural correlation with the same item residing in EM, a process directed by operations in WM which search for and recognize the representation that matches the present stimulus to indicate if it is a repeated “target” image. In contrast to the small effect of age on HITs, CRs, and RTs, the much greater inter-individual variability is likely related to fundamental differences between individuals across the lifespan, though any relationship to differences indicative of pathological processes needs further study.

In MemTrax, if the stimulus is “new,” it increases the length of the list of items to be stored, quickly exceeding the capacity of STM, thus requiring transfer of the item information into EM. This transfer makes each encoding and repeat analysis more difficult as the number of unique items perceived increases from 1 to 25, and the number of possible intervening, distracting items increases to 49, many occurring as repeated items more than a minute later. This progressive challenge allows for assessment of a broad range of cognitive abilities and potentially very early detection of cognitive impairment as is now widely desired for detecting early AD (Ashford et al., 2022b; Mattke et al., 2023).

The modest change in instructions from traditional SDTs stipulating the target items before the test begins to those of MemTrax delivering a progressive accumulation of a large number of target items during the CRT places a great demand on STM. The effects of distraction and decay on processes that are usually accommodated and directed by operations in WM instead require substantial encoding in EM, even beyond traditional applications of CRTs. This modification alters task complexity and the demands placed on individual vigilance during that intervening period between initial stimulus presentations and repetitions. The likelihood of responses thus infers events that IPMs suggest occur in the brain (Broadbent, 1965; Raaijmakers and Shiffrin, 1992), and are consistent with extensive study of human memory (Malmberg et al., 2019; Mulligan et al., 2023). Thus, the precision of the MemTrax CRT and its challenge for STM performance allows it to provide a substantial evaluation of STM from relatively normal individuals to those with mild to moderate EM impairment, potentially even exceeding the challenge of substantially delay recall testing (Wearn et al., 2020).

Current state-of-the-art Neuropsychological tests frequently take over 2 h of face-to-face administration most still using paper and pencil (the classic Luria-Nebraska test requires 18 h), making them costly, instructionally complicated, and time-consuming. These assessments classically are divided into measures of intelligence, language, visuospatial, memory (a broad function), and executive function. The critical issue in Alzheimer’s disease is measurement of episodic memory, which is awkwardly assessed by verbal learning tests, drawing a clock, and drawing recall tests. And few neuropsychological test components are suitable for frequent longitudinal assessment. While computerized batteries have been developed, mostly based on implementation of traditional tests, there is no accepted CRT for clinical utilization. The MemTrax CRT is characterized by high measurement precision and addresses several limitations of the current neuropsychological tests. Measuring the effects of FAs and misses on RTs for hits during performance of MemTrax across the items of the tests, including the types of categories and number of intervening stimuli which can be standardized for users of different ages and cultural and genetic backgrounds, may be used to describe the mathematical performance of STM for measuring cognitive processes directed by operations in WM. However, some aspects of intelligence, processing speed, language and visuospatial function, and executive function are also reflected in MemTrax performance. Further, MemTrax provides the precision to define the transition from normal to abnormal processing. Measurement of these additional aspects of cognition are important when assessing for clinical phenomena like AD, and further development of MemTrax and its analysis may provide such information.

The simple application of the MemTrax CRT can be used to measure RT to stimuli when these neurological processes are at least modestly preserved in AD patients or individuals with mild impairment. The application of an RT measurement for simple versus complex choice to measure cognitive ability, to make decisions about differences between stimuli, a distinction that requires maintenance in STM during the interval between occurrences, shows a specific impairment in AD (Pirozzolo et al., 1981). The requirement of making a complex choice requires the individual to examine their intentional maintenance of that information by those operations in WM during this testing, another specific impairment of AD (Mahurin and Pirozzolo, 1993). Consequently, the MemTrax implementation of the CRT paradigm could be of considerable utility for early detection and identification of the dementia associated with AD when the cognitive impairment is still mild. By precisely assessing the capability of the brain’s neural processing systems related to EM, the MemTrax test can be used for tracking later mild states with considerable precision as the AD pathology alters learning, memory and cognitive functions. The MemTrax metrics may also have utility in identifying clinical populations that may benefit from disease modifying or progression-prevention treatments. Further, the specific MemTrax metrics can define phenomena in target populations that can be applied to assess beneficial aspects of new treatments. By using MemTrax, an engaging assessment tool, the test itself will enhance enrollment and adherence to protocols in individuals who are likely to benefit from intervention. Provided over the time-course of a study of essentially any length, MemTrax can provide consistent assessment of the effectiveness of those treatments, thus increasing the power of study designs.

In addition, MemTrax may be used for patients with dementia who are presently unrecognized in the primary care setting. In 2011, Medicare added detection of cognitive impairment to its annual wellness visit.^3^ The Centers for Medicare and Medicaid Services’ guidance recommends assessing a patient’s learning, memory and cognitive functions by direct observation, including consideration of information and concerns reported by the patient, family members, friends, caregivers, and others, and, if appropriate, using a brief validated, structured assessment tool to objectively measure these functions (Masuda et al., 2022). MemTrax could provide data to the clinician for use in the objective evaluation of impairments expressed by the patient or caregiver during the in-office or virtual healthcare interview and for directing application of additional testing (Ashford et al., 2022b). The MemTrax results can then be evaluated and used by the clinician to increase certainty of diagnosis and to anticipate problems patients may have in understanding and adhering to medical treatment and plans needed for advance planning by patients and families. For a CRT like MemTrax to become a standard in an environment, for which it is well-suited, additional clinical work will be required to establish benchmarks across the continuum from the diverse normal population through the mild phases of memory dysfunction associated with early AD to the mild and moderate phases of dementia (Ashford and Schmitt, 2001; Ashford et al., 2023). However, the percentiles, SDs, and *Z*-scores provided in the tables are essentially identical across MemTrax studies, suggesting that this CRT is ready for clinical testing.

### Limitations and strengths

The HAPPYneuron data were based on population recruitment of anyone interested in online computer games that might preserve their memory. There was no population-based sampling and older individuals with significant memory difficulties who were not aware of their problems would not be likely to have become participants. Therefore, the level of cognitive impairment of any individual/participant was not determined. This selection process thus did not address this critical issue for validating the use of MemTrax as a cognitive assessment tool for the elderly. However, with so many individuals, the results presented here did represent a broad population of online users who were concerned about their memory. Further, much larger samples have been accumulated in other studies, including www.memtrax.com since 2013 (Ashford et al., 2022a), www.brainhealthregistry.org (Cholerton et al., 2019), and China (Liu et al., 2021), including two studies which have shown MemTrax to be at least as clinically useful a measure as the Montreal Cognitive Assessment (Van der Hoek et al., 2019; Liu et al., 2021). However, in-depth, case-controlled, clinical utilization studies are needed to develop precise metrics for screening for memory impairment and other cognitive functions and accurately and precisely assessing early dementia changes over time (Ashford et al., 2022b).

## Data availability statement

The raw data supporting the conclusions of this article will be made available by the authors, without undue reservation.

## Ethics statement

The studies involving humans were approved by the Stanford University Internal Review Board. The studies were conducted in accordance with the local legislation and institutional requirements. The ethics committee/institutional review board waived the requirement of written informed consent for participation from the participants or the participants’ legal guardians/next of kin because the data collected was anonymous, online.

## Author contributions

JC: Conceptualization, Data curation, Writing – original draft, Writing – review & editing. SA: Conceptualization, Methodology, Writing – review & editing. FT-B: Conceptualization, Data curation, Investigation, Methodology, Writing – review & editing. MB: Conceptualization, Investigation, Writing – review & editing. CA: Conceptualization, Funding acquisition, Methodology, Resources, Software, Writing – review & editing. PB: Conceptualization, Formal analysis, Writing – original draft, Writing – review & editing. JA: Conceptualization, Data curation, Methodology, Supervision, Writing – original draft, Writing – review & editing.

## Footnotes

1 https://www.cdc.gov/nchs/fastats/leading-causes-of-death.htm – Provisional Leading Causes of Death for 2023 - CDC Wonder- accessed 3/26/2024

2
www.memtrax.com

3
www.cms.gov/cognitive
